# DLX5 and HOXC8 enhance the chondrogenic differentiation potential of stem cells from apical papilla via LINC01013

**DOI:** 10.1186/s13287-020-01791-8

**Published:** 2020-07-06

**Authors:** Haoqing Yang, Yangyang Cao, Jianpeng Zhang, Yuncun Liang, Xiaomin Su, Chen Zhang, Huina Liu, Xiao Han, Lihua Ge, Zhipeng Fan

**Affiliations:** 1 Beijing Key Laboratory of Tooth Regeneration and Function Reconstruction, Beijing Stomatology Hospital, School of Stomatology, Capital Medical University, No. 4 Tian Tan Xi Li, Dongcheng District, Beijing, 100050 China; 2 Department of Endodontics, Beijing Stomatological Hospital, School of Stomatology, Capital Medical University, Beijing, 100050 China

**Keywords:** DLX5, HOXC8, Chondrogenic differentiation, Stem cells from apical papilla (SCAPs), LncRNA

## Abstract

**Background:**

Mesenchymal stem cell (MSC)-based cartilage tissue regeneration is a treatment with great potential. How to enhance the MSC chondrogenic differentiation is a key issue involved in cartilage formation. In the present study, we seek to expound the phenotypes and mechanisms of DLX5 in chondrogenic differentiation function in MSCs.

**Methods:**

Stem cells from apical papilla (SCAPs) were used. The Alcian Blue staining, pellet culture system, and cell transplantation in rabbit knee cartilage defect were used to evaluate the chondrogenic differentiation function of MSCs. Western blot, real-time RT-PCR, and ChIP assays were used to evaluate the molecular mechanisms.

**Results:**

DLX5 and HOXC8 expressions were upregulated during chondrogenic differentiation. In vitro results showed that DLX5 and HOXC8 enhanced the expression of chondrogenic markers including collagen II (COL2), collagen V (COL5), and sex-determining region Y box protein 9 (SOX9) and promoted the chondrogenic differentiation and the formation of cartilage clumps in the pellet culture system. Mechanically, DLX5 and HOXC8 formed protein complexes and negatively regulated the LncRNA, LINC01013, via directly binding its promoter. In vivo transplantation experiment showed that DLX5 and HOXC8 could restore the cartilage defect in the rabbit knee model. In addition, knock-down of LINC01013 enhanced the chondrogenic differentiation of SCAPs.

**Conclusions:**

In conclusion, DLX5 and HOXC8 enhance the chondrogenic differentiation abilities of SCAPs by negatively regulating LINC01013 in SCAPs, and provided the potential target for promoting cartilage tissue regeneration.

## Introduction

As a hypocellular and hypovascular tissue, the articular cartilage is formed by a specific extracellular matrix surrounding rare chondrocytes, and defects caused by natural degeneration or trauma may cause irreversible damage to its structure and function, which are leading sources of disability worldwide [1]. Conventional treatment strategies such as abrasion arthroplasty, bone marrow-stimulating repair, arthrocentesis, microfracture, and arthroscopic debridement have been used to solve this problem [2]. However, these strategies lack therapeutic efficacy owing to the low efficiency in restoring normal organization and function of the cartilage. Thus, evaluation of new and effective strategies for cartilage regeneration is necessary to establish a viable treatment plan. Mesenchymal stem cell (MSC)-mediated articular cartilage tissue regeneration is considered a promising method for cartilage injury treatment. Nowadays, regeneration of the cartilage in the joint based on MSCs has made significant progress [3–5]. MSCs are multipotent cells that were initially isolated from the bone marrow. Increasing evidence suggests that MSCs are multipotent, exhibit multiple differentiation potential, and could self-renew, so they are a reliable cell source for tissue regeneration. In the meanwhile, research has confirmed that a new type of MSCs was isolated from dental tissue (non-bone marrow tissue). These dental MSCs include dental pulp stem cells (DPSCs), stem cells from human exfoliated deciduous teeth (SHEDs), periodontal ligament stem cells (PDLSCs), and stem cells from apical papilla (SCAPs) [6]. They exhibit strong differentiation potential and self-renewal ability [7, 8]. Studies on the chondrogenic lineage characteristics of dental MSCs have also been done [9–12]. In particular, SCAPs showed a higher growth rate and more energetic osteo/odontogenic potential than PDLSCs and DPSCs [10]. The superior differentiation capability of SCAPs positions them as a promising alternative seeded cell for MSC-based tissue regeneration. Besides, other studies have reported that SCAPs also have strong chondrogenic differentiation potential [8, 13]. Despite these reports, a clear differentiation mechanism of MSCs is essential for adequately utilizing the potential of MSCs via inducing pluripotent stem cells to differentiate toward specific cell types or tissue [14]. Moreover, adequate recognition of the lineage and cell fate decision is necessary to generate efficient directed differentiation [15].

Chondrogenic progenitors are characterized by significantly high expression of Hox genes, strongly upregulated during limb formation and morphogenesis [16]. Previous studies showed that Dlx5 may play a decisive role in regulating the differentiation and maturation of chondrocytes [17]. Distal-less (Dlx) genes belong to the HOX family, which presented in *Drosophila*, and the same gene system conserved in humans and mice called the DLX gene family [18–20]. Dlx5, as a member of the transcription factor homologous domain family, plays pivotal roles during embryonic development and cell differentiation. In vivo studies suggest that Dlx5 is one of the earliest genes expressed in condensing the limb mesenchyme, which will give rise to the limb skeleton [21, 22]. Later, more mature cartilage is present in the perichondrium/periosteum in the extremities as well as the ribs and vertebrae [21, 23, 24]. Examination of the limbs of Dlx5 mutant embryos and neonates reveals an inconspicuous defect in chondrocyte hypertrophy which led to a delay in chondrocyte maturation [22], while studies have shown that knockout of both Dlx5 and Dlx6 genes caused more obvious deficiencies in chondrocyte hypertrophy [25]. Similarly, the silencing of the Dlx5 genes in chicken embryo results in ectopic chondrocyte hypertrophy which leads to the formation of severely shortened skeletal elements [26]. Previous reports also reveal that Dlx5 plays an essential role in the regulation of chondroblast marker genes during chondrogenic differentiation [26]. Over-expression of Dlx5 enhances early and late chondrocyte differentiation as well as osteoblast differentiation during endochondral ossification and inhibits proliferation in cultured cells in vitro [21, 22, 26]. Currently, the function of DLX5 in directed chondrogenic differentiation of human MSCs is unknown.

It has been postulated that DLX5 associate with HOXC8 to form a protein complex [27]. HOXC8 regulates the proliferation of chondrocytes as well as promotes cartilage maturation and endochondral ossification [28, 29]. Abnormal expression of HOXC8 causes cartilage defects as a result of the accumulation of proliferating chondrocytes and reduced maturation [30]. However, the role of HOXC8 and its relationship with DLX5 in chondrogenic differentiation of dental MSC remain unclear.

Long non-coding RNAs (lncRNAs, > 200 bp) are long non-translational RNAs involved in many pivotal biological processes. They play important roles in the development and progression of chondrogenesis [31]. For instance, lncRNA-HIT are deemed as an indispensable factor for mouse chondrogenic differentiation in the limb mesenchyme [31]. In addition, other lncRNAs such as zbed3-as1, ROCR, and UCA1 can target and regulate the formation of cartilage tissue [32]. Despite their invaluable roles in chondrogenic differentiation, the actual regulation mechanism that aids them to promote chondrogenic differentiation is unknown.

Herein, SCAPs were used to investigate the role and mechanism of DLX5 and HOXC8 in chondrogenic differentiation. The results of our research show that DLX5 and its partner HOXC8 could promote the chondrogenic differentiation of SCAPs. And we also identified their downstream target gene LINC01013 and regulating mechanism.

## Materials and methods

### Cell culture

SCAPs were gently isolated from the apical papillae of immature third molars obtained from patients in the Beijing Stomatological Hospital of the Capital Medical University. The patients gave an informed consent prior to the study. The apical papilla tissues were first covered in a solution comprising 3 mg/ml collagenase type I (Invitrogen, Carlsbad, CA, USA) and 4 mg/ml dispase (Invitrogen) for 60 min at 37 °C. Single-cell suspensions were then obtained using a 70-mm strainer (Falcon, BD Labware, Franklin Lakes, NJ, USA). The suspensions were incubated in DMEM alpha modified Eagle’s medium (Invitrogen, Carlsbad, CA, USA) supplemented with 15% fetal bovine serum (FBS; Invitrogen), 200 mM l-glutamine, and 10,000 units/ml penicillin-streptomycin (Invitrogen) in an incubator set at 37 °C and 5% carbon dioxide. We used flow cytometry to identify stem cells, and the results were showed in our previous article [33]. The suspensions were transferred to a fresh cell culture medium every 3 days.

Human embryonic kidney 293T (HEK 293T, American Type Culture Collection, Manassas, VA, USA) cells were incubated in complete DMEM supplemented with 10% FBS (Invitrogen), 100 U/ml penicillin, and 100 μg/ml streptomycin (Invitrogen). The 293T cells were used to package viral constructs.

### Alcian Blue staining and quantitative analysis

Chondrogenic differentiation was induced by using the StemPro chondrogenesis differentiation kit (Invitrogen). SCAPs were seeded onto 6-well plates (Costar) containing chondrogenic medium at 2.0 × 10^5^ cells/well to examine the chondrogenic differentiation potential. The medium was changed every 3 days. MSCs were grown in the chondrogenic medium for 3 weeks. For Alcian Blue staining, cells were fixed with 4% formaldehyde solution at room temperature for 60 min and then rinsed thoroughly with PBS. After that, cells were dyed in the 1% Alcian Blue solution for 30 min. Then, wells were rinsed three times with 0.1 N HCl to remove unstained areas, and distilled water was used to neutralize the pH value. The stained cells were observed under an inverted biological microscope, and images were captured for analysis. For quantitative analysis, the staining was dissolved with 300 μl of 6 M GuHCl for 12 h. The extract was measured by spectrophotometer (Molecular Devices) at 620 nm.

### Plasmid construction and viral infection

The plasmids used in this study were constructed with standard protocols; all constructs were verified by gene sequencing. The cDNA of human DLX5 or HOXC8 were subcloned into the LV5 lentiviral vector (Genepharma Company, Suzhou, China) separately for over-expression in SCAPs. Short hairpin RNAs (shRNAs) of DLX5, LINC01013, or HOXC8 was subcloned into the LV3 lentiviral vector (Genepharma). Then, SCAPs were plated and cultured for overnight in 100-mm dish and infected with lentiviruses for 12 h, containing polybrene (6 μg/ml, Sigma-Aldrich, St. Louis, MO, USA). The fresh medium was replaced after 48 h, and the transfected SCAPs were selected with the appropriate antibiotics. The target sequences for the shRNAs were as follows: HOXC8 shRNA (HOXC8sh), 5′-GGAGACGCCTCCAAATTCTAT-3′; DLX5 shRNA (DLX5sh), 5′-GTGCAGCCAGCTCAATCAA-3′; LINC01013 shRNA (LINC01013sh), 5′-GGTAATGACTGAGGTTATTCC-3′; and control shRNA (Consh), 5′-TTCTCCGAACGTGTCACGTTTC-3′.

### Pellet culture system and histological examination

To form a cell pellet, 2 × 10^5^ cells/ml centrifuged in 15 ml polypropylene conical tube at 1100 rpm for 6 min. Pellets were incubated in the StemPro chondrogenesis differentiation medium at 37 °C, 5% CO_2_ for 21 days. The culture medium was changed every 3 days. Primary cell pellets were fixed in 4% PFA (m/v) for 24 h and embedded in paraffin. Cell pellet sections were stained with the Alcian Blue Stain Kit (Cat No. G2541, Solarbio, Beijing, China) and Picro Sirius Red Stain Kit (Cat No. G1470, Solarbio). For Alcian Blue staining, specimens were treated with 3% acetic acid (pH 2.5) for 5 min and stained with 1% Alcian Blue for 1 h before counter-staining with nuclear fast red for 5 min. For Picro Sirius Red staining, the sections were incubated in 0.1% of Sirius Red in saturated aqueous picric acid for 1 h and rinsed with hydrochloric acid (0.01 M) for 2 min.

### Co-immunoprecipitation (Co-IP) assay

Cells were collected and lysed with the IP lysis buffer (Invitrogen) and protease inhibitor cocktail (Roche) and centrifuged at 14000*g* for 10 min at 4 °C. Cell lysates were incubated with specific primary antibody for 4 h and then added protein A/G Sepharose (Santa Cruz) overnight at 4 °C. The sepharose beads were washed with lysis buffer three times and resuspended in SDS-PAGE loading buffer for Western blotting analysis using corresponding antibodies. The primary antibodies were as follows: DLX5 (Clone No.1B7, Cat No. PAB13670, Abnova, Taipei City, Taiwan) and HOXC8 (Clone No.1H2, Cat No. H00003224-M02, Abnova).

### Western blot analysis

The protein extraction and the gel electrophoresis tests were performed as previously described [34]. The primary antibodies were as follows: DLX5 (Clone No.1B7, Cat No. PAB13670, Abnova), HOXC8 (Clone No.1H2, Cat No. H00003224-M02, Abnova), and a monoclonal antibody specific for the house-keeping protein, glyceraldehyde 3-phosphate dehydrogenase (GAPDH; Clone No.GAPDH-71.1, Cat No.G8795, Sigma-Aldrich).

### Real-time reverse transcriptase PCR

Total RNA was isolated from SCAPs with TRIzol Reagent (Invitrogen). Then, 2-μg aliquots of RNA with oligo (dT) or random primers and reverse transcriptase were synthesized cDNA, according to the manufacture’s protocol (Invitrogen). Real-time PCR reactions were performed with the QuantiTect SYBR Green PCR kit (Qiagen, Hilden, Germany) and an Icycler iQ Multicolor Real-time PCR Detection System. Each reaction was run in triplicate, and the entire procedure was repeated three times. The primers used are shown in Table S1.

### Cartilage defect established in rabbit knee and SCAP transplant experiment

All animals were treated according to the animal care and following the animal experiment ordinance of the Beijing Stomatological Hospital, Capital Medical University. The care and use of animals were performed according to the guidelines of the Experimental Animal Management Ordinance. We used nine New Zealand rabbits (6 months old, 3–3.5 kg) to establish knee cartilage defects and randomly divided into 3 groups (SCAP/Vector, SCAP/DLX5 group, and SCAP/HOXC8 group); each group has 6 knee cartilage defects in 3 rabbits. Then, we used 3 New Zealand rabbits (6 months old, 3–3.5 kg) as the sham group, which were only open the joint capsule and then stitched it together. The 3% pentobarbital sodium (35 mg/kg) was used for intravenous anesthesia in rabbits. The rabbits’ knee joints were opened with a medial parapatellar approach. A cylindrical full-thickness osteochondral defect (4 mm diameter × 4 mm depth) was drilled in the medial femoral condyle using a standard 4-mm hollow drill [35, 36]. A mixture of 1 × 10^6^ cells and 50 μl of Matrigel® Matrix (Cat No. 356234, Corning, USA) was then slowly injected into the defect site. The vector group (SCAP/Vector with Matrigel), DLX5 group (SCAP/DLX5 with Matrigel), and HOXC8 group (SCAP/HOXC8 with Matrigel) were applied. And the sham group was used as normal control. The mixture was left to solidify prior to implantation. After implantation, the patella and femoral were repositioned and sutured layer by layer with absorbable sutures. Intramuscular injection of penicillin (100,000 U/kg) was given to each rabbit for 7 days to prevent infection. All animals were killed 12 weeks after surgery.

### Histological examination of the repaired cartilage

All samples were harvested, fixed in 10% formalin, and decalcified in 10% EDTA. The tissues were then sliced into 5-μm sections and stained with hematoxylin and eosin (H&E). The newly formed cartilage and cartilaginous matrix were examined using commercial kits for Alcian Blue staining (Cat No. G2541, Solarbio), Safranin-O staining (Cat No. G1470, Solarbio), and Toluidine Blue staining (Cat No. G3661, Solarbio). The expression of COL-2 in the tissue sections was determined using immunohistochemical staining assays by COL-2 antibody (Cat No. bs-10589R, Bioss, Beijing, China). The degree of tissue regeneration was graded in a blinded manner by 5 graders according to the International Cartilage Repair Society (ICRS) scoring system as described previously [37, 38].

### Chromatin immunoprecipitation (ChIP) assays

The ChIP assay was performed as previously described using a kit (Merck Millipore, Darmstadt, Germany) [34]. Anti-HOXC8 antibody (Clone No.1H2, Cat No. H00003224-M02, Abnova) was used for precipitation. The precipitated DNA samples were quantified with real-time RT-PCR. Data are expressed as a percentage of input DNA. The primer sequences were as follows: LINC01013 promoter binding site 1 (LINC01013-BS1), forward 5′-AGGTACACGCATCCTCCCTA-3′ and reverse 5′-TCCATATCCGCAGTTCCACAC-3′; LINC01013 promoter binding site 2 (LINC01013-BS2), forward 5′-ACAGTGTGTCAGCAGCCAAG-3′ and reverse 5′-TTCCTACAAGTTGCCCAGCT-3′; LINC01013 promoter binding site 3 (LINC01013-BS3), forward 5′-AGCTGGGCAACTTGTAGGAA-3′ and reverse 5′-GCCAGTAGAAATGCAGCCAC-3′; and negative control (5 kb down, 5 kb downstream of LINC01013 promoter binding site 3), forward 5′-GGTGGAGCAGGAGAGAGAGA-3′ and reverse 5′-GGGCAGATTTCCTCCTTGCT-3′.

### Statistical analyses

Statistical analyses were performed using the SPSS version 22 statistical software. In vitro experiments were independently repeated three times. For in vivo experiments, six samples were used in each group for statistical analyses. The statistical significance was determined using Student’s *t* test or one-way ANOVA analysis, and a *p* ≤ .05 was considered significant.

## Results

### DLX5 enhanced the chondrogenic differentiation potential of SCAPs

**Fig. 1:**
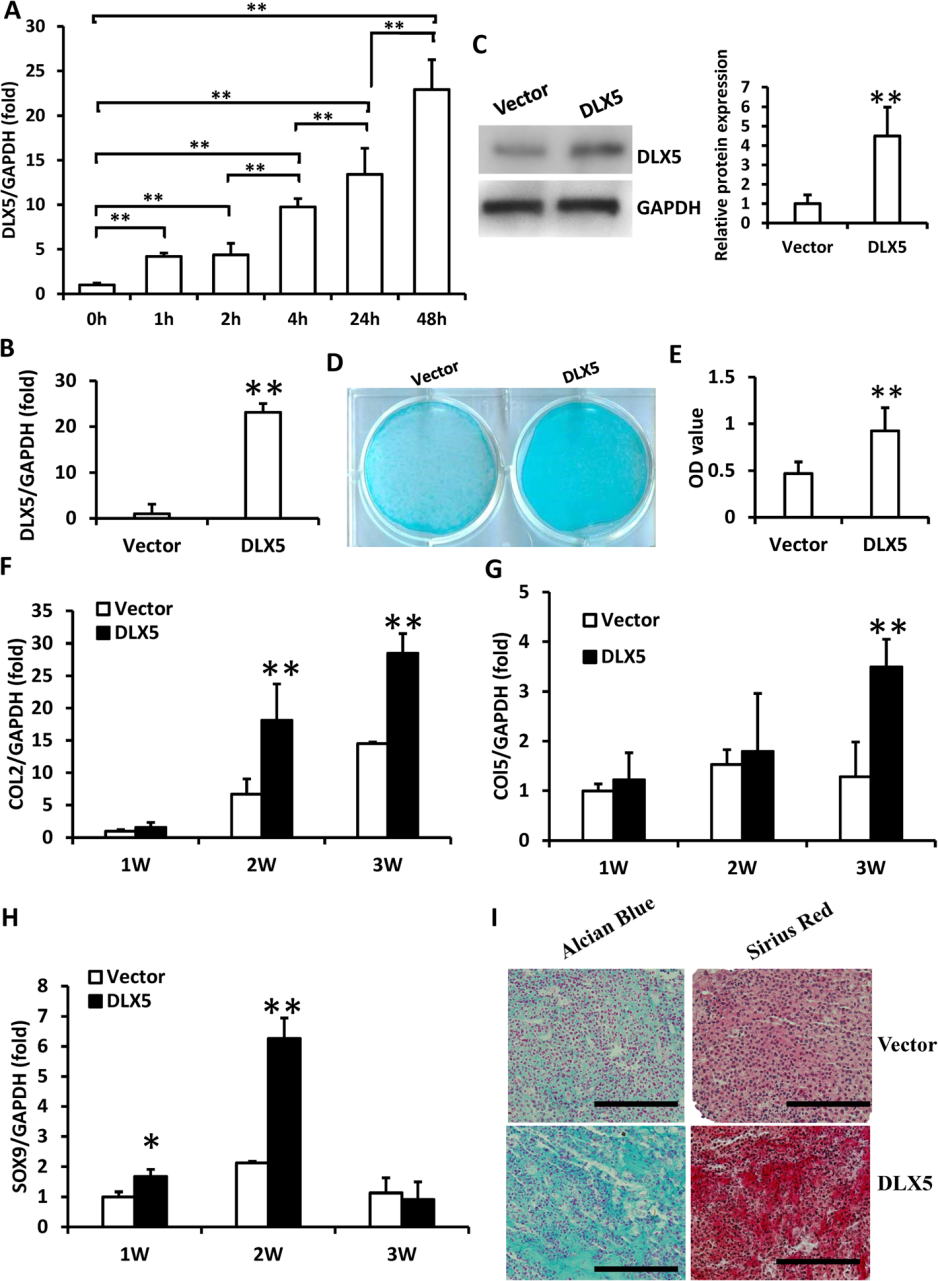
DLX5 over-expression enhanced the chondrogenic differentiation in SCAPs. **a** DLX5 expression during chondrogenic differentiation as detected by real-time RT-PCR. **b**, **c** Real-time RT-PCR and Western blot results showed the DLX5 expression in SCAPs. The relative protein levels quantified by densitometry and normalized to GAPDH. **d**, **e** Alcian Blue staining and quantitative analysis results show that DLX5 over-expression enhanced chondrogenic differentiation in SCAPs. **f**–**h** Real-time RT-PCR results show that DLX5 over-expression upregulated the expression of COL2 (**f**), COL5 (**g**), and SOX9 (**h**) in SCAPs. **i** Alcian Blue and Picro Sirius Red staining results of chondrogenesis induced pellet. Scale bar, 100 μm. GAPDH was used as an internal control. One-way ANOVA or Student’s *t* test analysis was performed to determine the statistical significance. All error bars represent s.d. (*n* = 3). *P ≤ 0.05. **P ≤ 0.01

The expression of DLX5 was examined at the early stage of chondrogenic differentiation in SCAPs. The real-time RT-PCR revealed that DLX5 expression was upregulated at 1, 2, 4, 24, and 48 h after chondrogenic induction in SCAPs (Fig. 1a). Then, SCAPs were transduced with DLX5 and empty vector. In order to purify the infected cells, the transduced SCAPs were treated with 2 μg/ml puromycin for 3 days. The real-time RT-PCR and Western blot results confirmed the ectopic DLX5 over-expression (Fig. 1b, c). SCAPs transduced with DLX5 and empty vector were cultured in chondrogenic-inducing medium to determine the chondrogenic differentiation potential. At 3 weeks after induction, the Alcian Blue staining and quantitative analysis results showed that glycosaminoglycan formation was enhanced in SCAPs infected with DLX5 than in the control cells (Fig. 1d, e). Real-time RT-PCR results showed that the chondrogenic differentiation markers were changed, including collagen II (COL2) expression was upregulated at 2 and 3 weeks, collagen V (COL5) expression was upregulated at 3 weeks, and sex-determining region Y box protein 9 (SOX9) expression was upregulated at 1 and 2 weeks after chondrogenic induction in SCAPs infected with DLX5 compared to control cells (Fig. 1f–h). Moreover, in order to investigate the role of DLX5 on chondrogenesis, the SCAPs were induced to form cartilage pellets in in vitro culture system. The Alcian Blue and Picro Sirius Red staining results showed that the pellets which formed by DLX5 over-expressed SCAPs showed higher chondrogenesis potential than the control group at 3 weeks after induction (Fig. 1i).

**Fig. 2:**
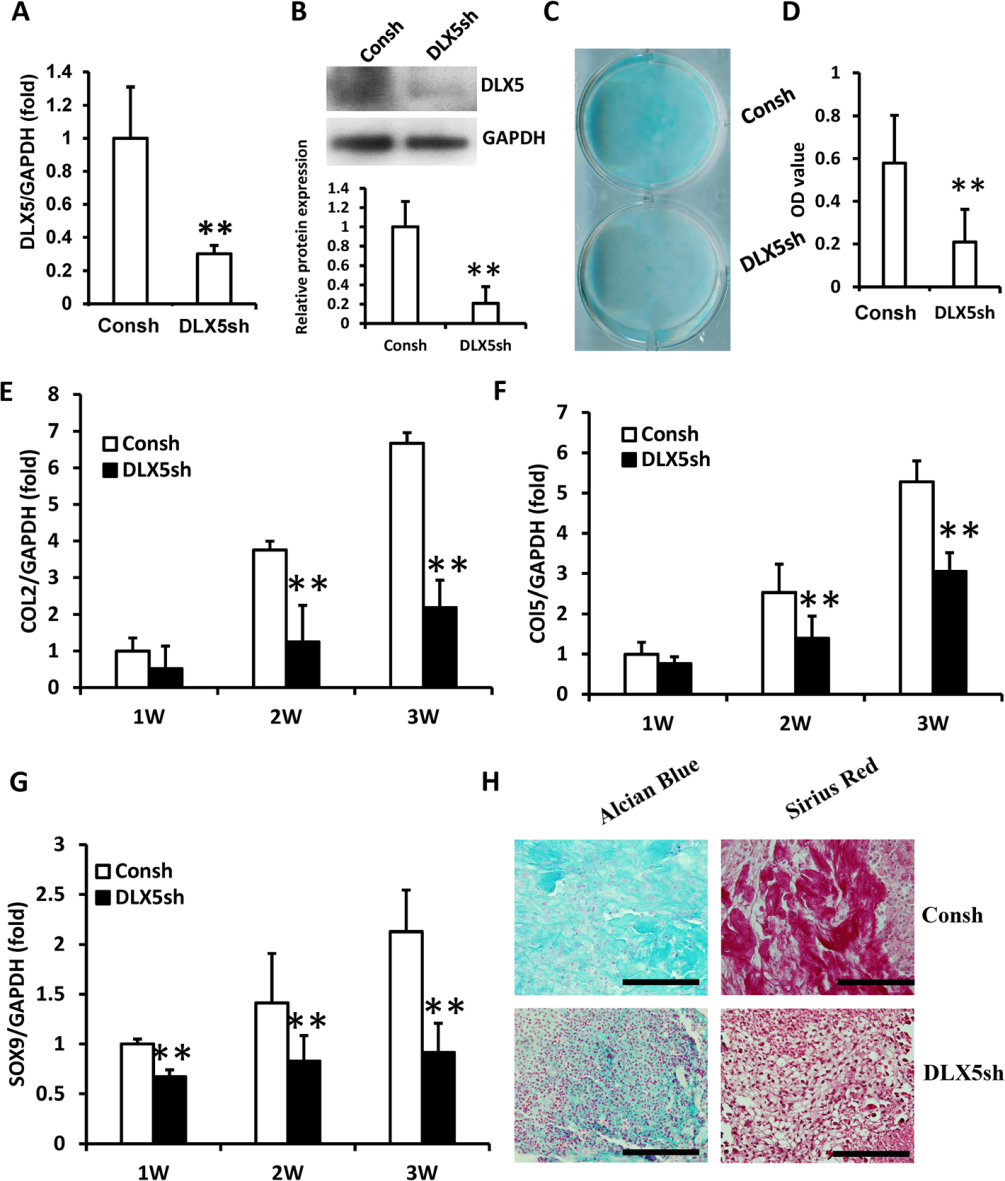
DLX5 knock-down inhibited the chondrogenic differentiation in SCAPs. **a**, **b** Real-time RT-PCR and Western blot results showed that DLX5 was knocked down in SCAPs. The relative protein levels quantified by densitometry and normalized to GAPDH. **c**, **d** Alcian Blue staining and quantitative analysis results show that knock-down of DLX5 reduced chondrogenic differentiation in SCAPs. **e**–**g** Real-time RT-PCR results show that knock-down of DLX5 downregulated the expressions of COL2 (**e**), COL5 (**f**), and SOX9 (**g**) in SCAPs. **h** Alcian Blue and Picro Sirius Red staining results of chondrogenesis induced pellet. Scale bar, 100 μm. GAPDH was used as an internal control. Student’s *t* test was performed to determine the statistical significance. All error bars represent s.d. (*n* = 3). **P ≤ 0.01. Consh, control shRNA; DLX5sh, DLX5 shRNA.

To further discover the function of DLX5 in chondrogenic differentiation of SCAPs, the expression of DLX5 was inhibited by using lentivirus-mediated DLX5 shRNA. In order to purify the infected cells, the transduced SCAPs were treated with 2 μg/ml puromycin for 3 days. The real-time RT-PCR and Western blot results revealed that DLX5 was efficiently silenced in SCAPs (Fig. 2a, b). At 3 weeks after induction, both Alcian Blue staining and quantitative analysis results showed that glycosaminoglycan formation was inhibited in DLX5-depleted SCAPs than in the control cells (Fig. 2c, d). Then, the real-time RT-PCR results showed that COL2 and COL5 expressions were decreased at 2 and 3 weeks, and SOX9 expression was decreased at 1, 2, and 3 weeks after chondrogenic induction in DLX5-depleted SCAPs compared to the control group (Fig. 2e–g). At 3 weeks after cartilage pellet culture, the Alcian Blue and Picro Sirius Red staining results showed that the pellets which formed by DLX5-depleted SCAPs showed less chondrogenesis potential compared with the control group (Fig. 2h).

### HOXC8 enhanced the chondrogenic differentiation potential of SCAPs

**Fig. 3:**
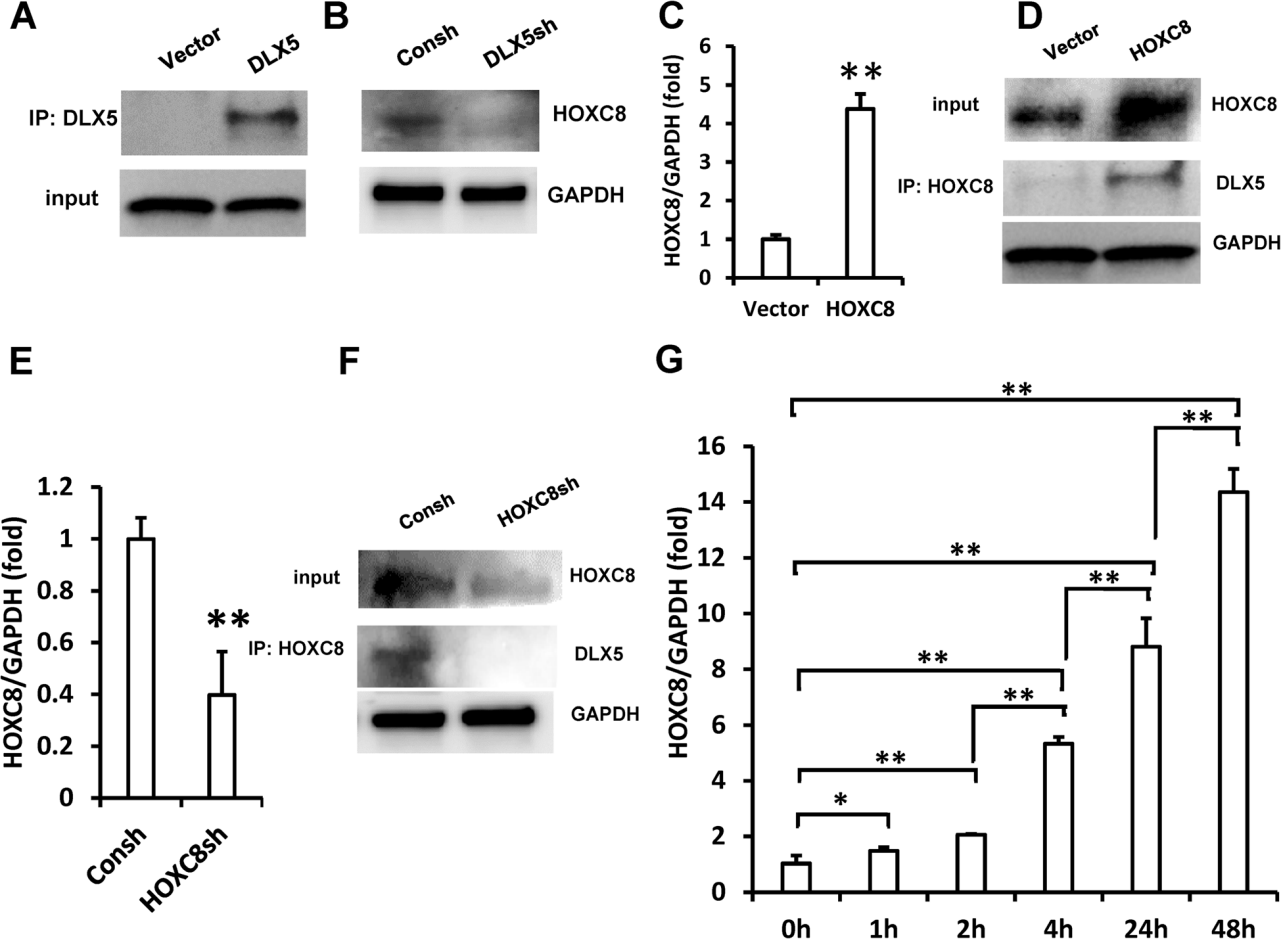
DLX5 associated with HOXC8 and formed protein complex in SCAPs. **a** Co-IP results showed more DLX5-HOXC8 complex formation in DLX5 over-expressed SCAPs **b** Co-IP results showed less DLX5-HOXC8 complex formation in DLX5-silenced SCAPs. **c** Real-time RT-PCR results showed HOXC8 over-expression in SCAPs. **d** Western blot results showed HOXC8 over-expression in SCAPs. Co-IP results showed more HOXC8-DLX5 complex formation in HOXC8 over-expressed SCAPs. **e** Real-time RT-PCR results show HOXC8 depletion in SCAPs. **f** Western blot results show HOXC8 depletion in SCAPs. Co-IP results showed fewer endogenous HOXC8-DLX5 protein complexes in HOXC8-silenced SCAPs. **g** HOXC8 expression was increased during the chondrogenic differentiation in SCAPs according to real-time RT-PCR results. GAPDH served as the internal control. One-way ANOVA or Student’s *t* test analysis was performed to determine the statistical significance. All error bars represent s.d. (*n* = 3). *P ≤ 0.05. ** *P* ≤ 0.01. Consh, control shRNA; DLX5sh, DLX5 shRNA; HOXC8sh, HOXC8 shRNA

The Co-IP assay was used to detect the possible binding partner of DLX5 in SCAPs; the results showed that over-expression of DLX5 could interact with more HOXC8 in SCAPs (Fig. 3a). Furthermore, the Co-IP results showed that DLX5 depletion inhibited the formation of HOXC8 and DLX5 protein complexes in SCAPs (Fig. 3b). The influence of over-expression or depletion of HOXC8 on the formation of protein complexes in SCAPs was further investigated. The HOXC8 sequence was inserted into a lentiviral vector and SCAPs infected with the lentivirus. The transduced SCAPs were treated with 2 μg/ml puromycin for 3 days to purify the infected cells. The real-time RT-PCR and Western blot results confirmed the ectopic HOXC8 over-expression in SCAPs (Fig. 3c, d). The Co-IP results showed that the over-expression of HOXC8 could recruit more DLX5 in SCAPs (Fig. 3d). Then, HOXC8 was inhibited by using lentivirus-mediated HOXC8 shRNA and the silencing efficiency confirmed by both real-time RT-PCR and Western blot analysis results (Fig. 3e, f). The Co-IP results showed that HOXC8 depletion inhibited the formation of HOXC8/DLX5 protein complexes in SCAPs (Fig. 3f). Next, we investigated the expression of HOXC8 at the early stage of chondrogenic differentiation in SCAPs. The real-time RT-PCR results showed that HOXC8 expression was upregulated at 1, 2, 4, 24, and 48 h after chondrogenic induction in SCAPs (Fig. 3g).

**Fig. 4:**
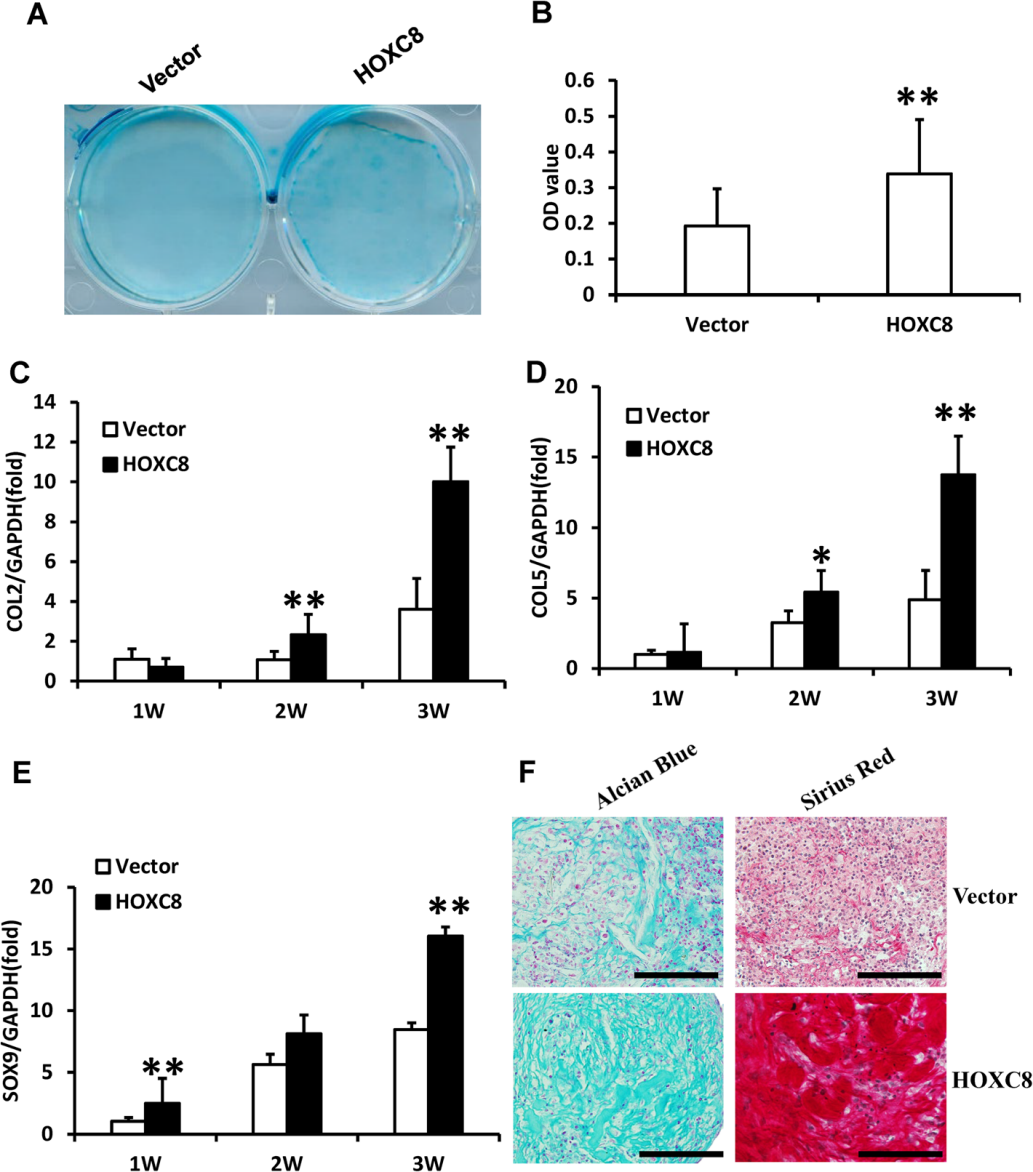
HOXC8 over-expression enhanced the chondrogenic differentiation of SCAPs. **a**, **b** Alcian Blue staining and quantitative analysis results showed that HOXC8 over-expression enhanced chondrogenic differentiation in SCAPs. **c**–**e** Real-time RT-PCR results showed that HOXC8 over-expression upregulated the expression of COL2 (**c**), COL5 (**d**), and SOX9 (**e**) in SCAPs. **f** Alcian Blue and Picro Sirius Red staining results of chondrogenesis induced pellet. Scale bar, 100 μm. GAPDH was used as an internal control. Student’s *t* test was performed to determine the statistical significance. All error bars represent s.d. (*n* = 3). **P ≤ 0.01

Then, we investigated the chondrogenic differentiation potential of HOXC8 in SCAPs. After being cultured in the chondrogenic-inducing medium at 3 weeks, both Alcian Blue staining and quantitative analysis results showed that glycosaminoglycan formation was enhanced in HOXC8-infected SCAPs compared to control cells (Fig. 4a, b). The real-time RT-PCR results showed that COL2 and COL5 expression were upregulated at 2 and 3 weeks, and SOX9 expression was upregulated at 1 and 3 weeks after chondrogenic induction in HOXC8-infected SCAPs (Fig. 4c–e). At 3 weeks after cartilage pellet culture, both Alcian Blue and Picro Sirius Red staining results showed that the pellets which formed by HOXC8 over-expressed SCAPs showed higher chondrogenesis potential compared with the control group (Fig. 4f).

**Fig. 5:**
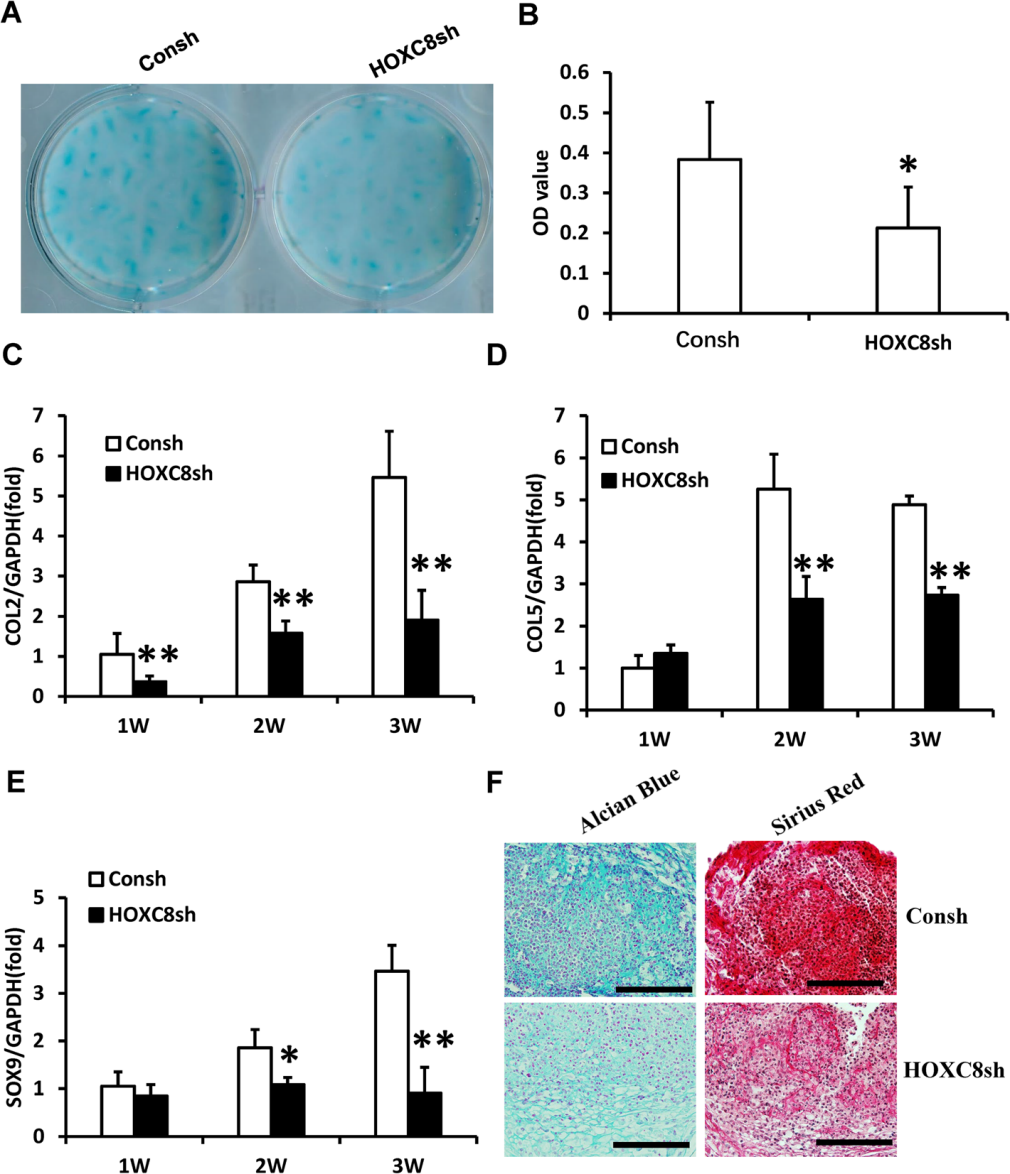
HOXC8 knock-down inhibited the chondrogenic differentiation of SCAPs. **a**, **b** Alcian Blue staining and quantitative analysis results showed that knock-down of HOXC8 reduced chondrogenic differentiation in SCAPs. **c**–**e** Real-time RT-PCR results showed that knock-down of HOXC8 downregulated the expressions of COL2 (**c**), COL5 (**d**), and SOX9 (**e**) in SCAPs. **f** Alcian Blue and Picro Sirius Red staining results of chondrogenesis induced pellet. Scale bar, 100 μm. GAPDH was used as an internal control. Student’s *t* test was performed to determine the statistical significance. All error bars represent s.d. (*n* = 3). *P ≤ 0.05. **P ≤ 0.01. Consh, control shRNA; HOXC8sh, HOXC8 shRNA.

The role of HOXC8 in chondrogenic differentiation of SCAPs was further investigated by silencing HOXC8. At 3 weeks after chondrogenic induction, both Alcian Blue staining and quantitative analysis results showed that glycosaminoglycan formation was inhibited in HOXC8 silenced SCAPs compared to the control group (Fig. 5a, b). The real-time RT-PCR results showed that COL2 expression was decreased at 1, 2, and 3 weeks, while COL5 and SOX9 expressions were decreased at 2 and 3 weeks after chondrogenic induction in HOXC8-silenced SCAPs compared to the control group (Fig. 5c–e). At 3 weeks after cartilage pellet culture, the Alcian Blue and Picro Sirius Red staining results showed that the pellets which formed by HOXC8-silenced SCAPs showed less chondrogenesis potential compared with the control group (Fig. 5f).

### DLX5 and HOXC8 enhanced SCAP-mediated cartilage regeneration in rabbit knee model

**Fig. 6:**
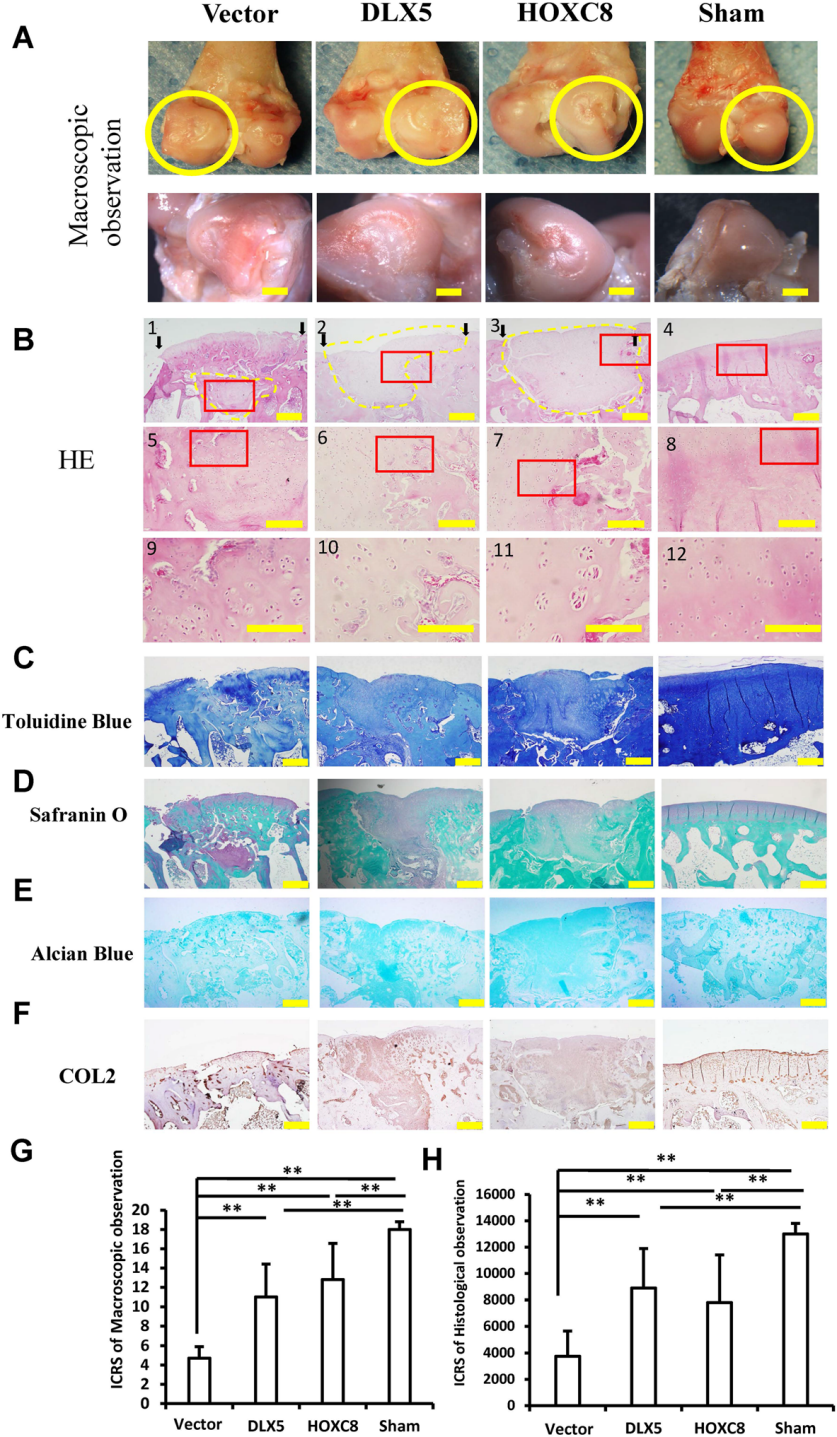
DLX5 and HOXC8 enhanced SCAP-mediated cartilage regeneration in rabbit knee models. **a** Macroscopic observation of cartilage defect healing. Scale bars, 1 mm. **b** HE staining results. Scale bars, 500 μm (B1–4), 200 μm (B5–8), and 100 μm (B9–12). (The black arrow shows the edge of the defect; B5–B8 is the partial enlargement of the red box in B1–B4; B9–B12 is the partial enlargement of the red box in B5–B8; the yellow dotted line shows the regenerated tissue at the defect site). **c** Toluidine Blue staining results. Scale bars, 500 μm. **d** Safranin-O staining results. Scale bars, 500 μm. **e** Alcian Blue staining results. Scale bars, 500 μm. **f** Immunohistochemical staining results of COL2. Scale bars, 500 μm. **g** Quantitative assessment of macroscopic results. **h** Quantitative assessment of histological results. One-way ANOVA analysis was performed to determine the statistical significance. All error bars represent s.d. (*n* = 6). **P ≤ 0.01

At 12 weeks after transplantation, the regenerated cartilage tissue was examined to compare the chondrogenesis effect of these genes. General observation results showed that the SCAP/Vector group had irregular surfaces with fibrosis and were incompletely healed. A visible defect was still present in the SCAP/Vector group, fissures in the defects were seen, the concavity was still noticeable, and the surfaces of tissues was less smooth than the SCAP/DLX5 and SCAP/HOXC8 groups (Fig. 6a). Compared with the SCAP/Vector group, the SCAP/DLX5 and SCAP/HOXC8 groups have better observations. The SCAP/DLX5 group appeared semi-transparently white, looked like a normal articular cartilage appearance, and there are depressions in the defect area (Fig. 6a). The SCAP/HOXC8 group appeared semi-transparently white with a yellowish cast and covered the articulating end of the femoral condyle and looked like a normal articular cartilage appearance. The reparative tissue in the wound appeared semi-transparent, and the margin was integrated with adjacent healthy tissue, the regenerated tissue almost fully filled in the defects (Fig. 6a).

The repaired tissues were further analyzed by histological examination. The HE staining results showed that in the SCAP/Vector group, the regenerated cartilage tissue was disordered, only the central part of the cartilage had formed a typical tissue structure, and the surface tissue was loose and porous (Fig. 6b). In the SCAP/HOXC8 and SCAP/DLX5 groups, there were large amounts of regenerated cartilage tissue in the defect area with a dense structure, which was not significantly different from the healthy tissue surrounding the defect area in color and texture. In addition, there are a large number of columnar cells deep in the regenerated tissue. The cartilage thickness and subchondral bone formation were similar to the original natural tissue. There was also a large amount of angiogenesis which could be seen at the boundary with the original tissue (Fig. 6b). The Toluidine Blue staining, safranin-O, and Alcian Blue staining results showed that cartilage-like tissues exist only on the surface and in the deep intermediate tissues in the SCAP/Vector group (Fig. 6c–e). However, in the SCAP/HOXC8 and SCAP/DLX5 groups, the defects were completely filled with cartilage-like tissue, which has good compatibility with the surrounding cartilage tissue; the content of the regenerated cartilaginous matrix was slightly lower than that of the normal cartilage (Fig. 6c–e). Immunohistochemical staining results revealed that there was a weak expression of COL2 on the tissue surface of rabbit knees in the SCAP/Vector group (Fig. 6f). However, the SCAP/HOXC8 and SCAP/DLX5 groups had a strong expression of COL2 at the site of the regenerated cartilage tissue (Fig. 6f). In the same line, the ICRS score of macroscopic results in the SCAP/HOXC8 and SCAP/DLX5 groups was much higher than that in the SCAP/Vector group (Fig. 6g). Similarly, the ICRS score of histological results in the SCAP/HOXC8 and SCAP/DLX5 groups was also much higher than that in the SCAP/Vector group (Fig. 6h).

### DLX5 and HOXC8 directly inhibited transcription of LINC01013 in SCAPs

**Fig. 7:**
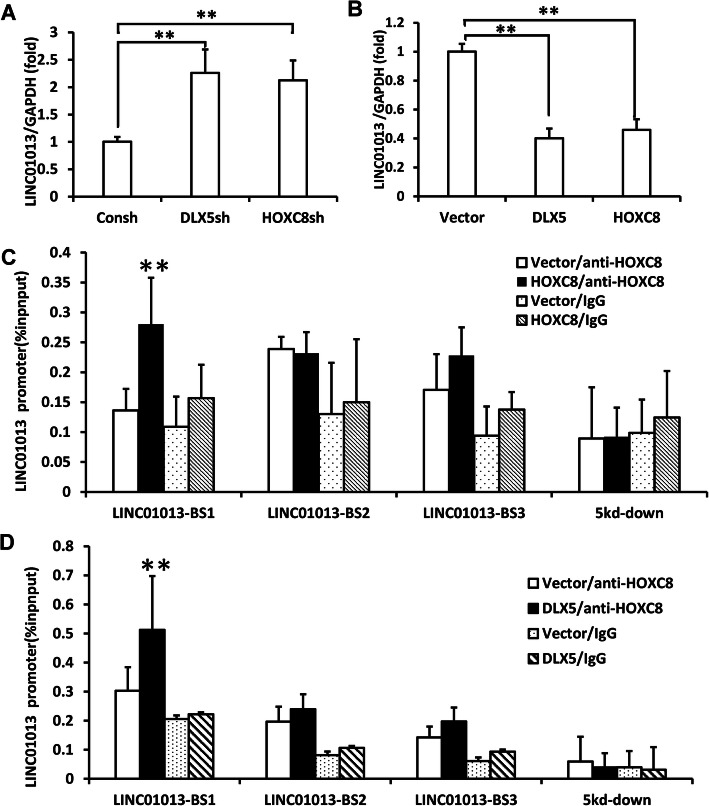
DLX5 and HOXC8 negatively regulated the LINC01013 by binding to the LINC01013 promoter in SCAPs. **a** LINC01013 was upregulated in DLX5 knocked down or HOXC8 knocked down SCAPs. **b** LINC01013 was downregulated in DLX5 over-expressed or HOXC8 over-expressed SCAPs. **c** ChIP assays showed HOXC8 over-expression enhanced the recruitment of HOXC8 into LINC01013 promoter in SCAPs. **d** ChIP assays showed DLX5 over-expression enhanced the recruitment of HOXC8 into LINC01013 promoter in SCAPs. GAPDH was used as an internal control. One-way ANOVA analysis was performed to determine the statistical significance. All error bars represent s.d. (*n* = 3). **P ≤ 0.01. Consh, control shRNA; DLX5sh, DLX5 shRNA; HOXC8sh, HOXC8 shRNA

Next, we wanted to find out whether DLX5 and HOXC8 work together during chondrogenic differentiation. We identified the target genes of HOXC8 and DLX5 that might be associated with its function by using RNA-seq analysis. The results showed that LINC01013 expression was upregulated after knock-down of HOXC8 and DLX5 (Fig. S1A). Next, the real-time RT-PCR results revealed that the knock-down of HOXC8 or DLX5 enhanced LINC01013 expression in SCAPs (Fig. 7a), and over-expression of HOXC8 or DLX5 repressed LINC01013 expression in SCAPs (Fig. 7b).

In addition, the bioinformatics analysis showed that there were abundant specific binding sequences of the HOX gene (TAAT/ATTA/TTAT/ATAA/TTAC) in the LINC01013 promoter, which might be identified and associated by HOXC8. The ChIP assay results showed that there were more HOXC8 proteins significantly associated with the region at 1987~1799 bp upstream of the LINC01013 promoter, which have several candidate HOXC8-binding elements in HOXC8 over-expressed SCAPs compared to the control group (Fig. S1B; Fig. 7c). Furthermore, ChIP assay results also showed that more HOXC8 proteins significantly associated with the HOXC8-binding site at the LINC01013 promoter in DLX5 over-expressed SCAPs compared to the control group (Fig. 7d).

### LINC01013 repressed the chondrogenic differentiation potential of SCAPs in vitro

**Fig. 8:**
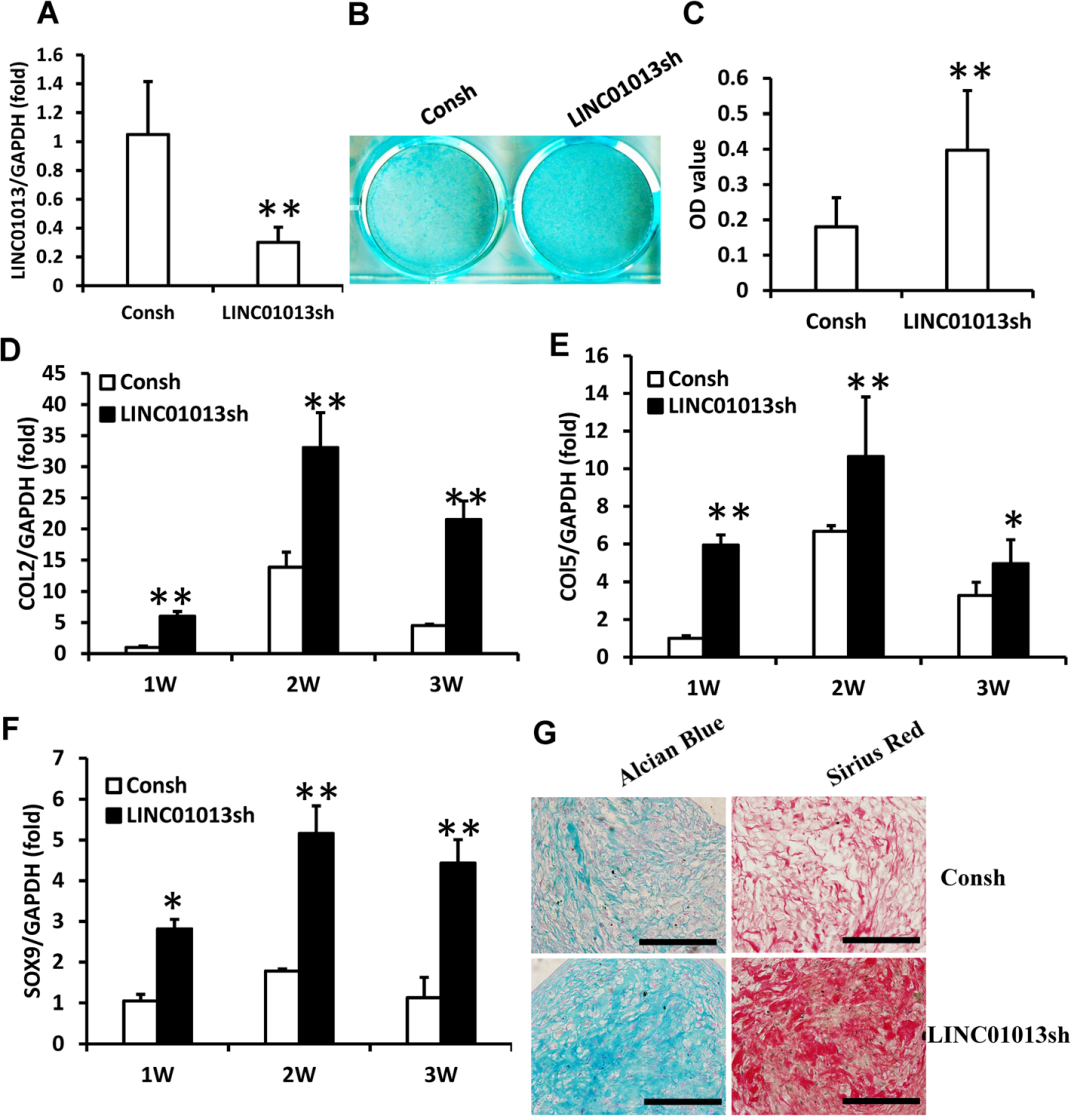
LINC01013 knock-down enhanced the chondrogenic differentiation in SCAPs. **a** Real-time RT-PCR results showed LINC01013 knock-down in SCAPs. **b**, **c** Alcian Blue staining and quantitative analysis results showed that knock-down of LINC01013 promoted chondrogenic differentiation in SCAPs. **d**–**f** Real-time RT-PCR results showed that knock-down of LINC01013 upregulated the expressions of COL2 (**d**), COL5 (**e**), and SOX9 (**f**) in SCAPs. **g** Alcian Blue and Picro Sirius Red staining results of chondrogenesis induced pellet. Scale bar, 100 μm. GAPDH was used as an internal control. Student’s *t* test was performed to determine the statistical significance. All error bars represent s.d. (*n* = 3). *P ≤ 0.05. **P ≤ 0.01. Consh, control shRNA; LINC01013sh, LINC01013 shRNA

To further illustrate the function of LINC01013 in chondrogenic differentiation of SCAPs, we inhibited LINC01013 expression by using lentivirus-mediated LINC01013 shRNA. In order to purify the infected cells, the transduced SCAPs were treated with 2 μg/ml puromycin for 3 days. The real-time RT-PCR results showed the knock-down efficiency of LINC01013 in SCAPs (Fig. 8a). Both Alcian Blue staining and quantitative analysis results showed that glycosaminoglycan formation was increased in LINC01013 knocked down SCAPs compared to control cells (Fig. 8b, c). Real-time RT-PCR results showed that COL2, COL5, and SOX9 expressions were increased at 1, 2, and 3 weeks after chondrogenic induction in LINC01013 knocked down SCAPs compared to control cells (Fig. 8d–f). At 3 weeks after cartilage pellet culture, the Alcian Blue and Picro Sirius Red staining results showed that the pellets which formed by LINC01013 knocked down SCAPs showed higher chondrogenesis potential compared with the control group (Fig. 8g).

## Discussion

Application of MSCs for cartilage tissue regeneration has interested numerous scholars. This has especially been the case owing to the advancements made in tissue engineering technologies. Furthermore, the regulation of directional differentiation of MSC is the core issue for enhancing MSC-mediated tissue regeneration. The bone marrow MSCs (BMSCs) have been used in most chondrogenic differentiation and regeneration studies. Autologous and allogeneic BMSC transplantation studies have shown that they can produce chondroblast cells and fibrocartilage in the defect areas [39, 40]. However, the application of BMSCs is restricted by two main problems: their low rate of chondrogenic differentiation and the variable maintenance time of chondrogenic differentiation [41]. Dental-derived MSCs are promising candidates for cartilage regeneration due to their high cartilage differentiation capability [9, 42]. They also have superior proliferation capacity and multipotent differentiation capacity than BMSCs [7, 43]. In addition, some studies indicate that dental MSCs have superior chondrogenic differentiation potential than BMSCs in vitro and in vivo [44–46]. These characteristics make dental MSCs a promising candidate seed cell in MSC-mediated therapies. So, it is thus necessary to clear the molecular mechanism of dental MSC-mediated chondrogenic tissue regeneration.

Herein, the capability of DLX5 in chondrogenic differentiation of MSCs derived from the apical papilla was investigated. DLX5 was upregulated during chondrogenic differentiation in SCAPs. DLX5 also promoted chondrogenic differentiation as well as increased the expression of chondrogenesis-related genes such as COL2, COL5, and SOX9 during chondrogenic differentiation. Extracellular matrix proteins (ECM) COL2 and COL5 are specific proteins in cartilaginous tissues that are essential for normal bone embryonic development and chondrocyte differentiation [47, 48]. SOX9 on the other hand is a transcription factor that is critical during cartilage formation [49, 50]. However, the expression of the chondrogenic marker, such as SOX9, was a dynamic change in the chondrogenic differentiation process, which may be the reason to explain the expression of SOX9 had no statistical difference after over-expression of DLX5 at 3-week time point.

Moreover, SOX9 expression is regulated by members of the Wnt, bone morphogenetic protein (BMP), FGF, and TGF-b families [51, 52]. BMP plays a pivotal role in chondrogenic development and chondrogenic differentiation [53]. Our previous study showed that DLX5 was regulated by BMP signaling via canonical SMAD signaling [34]. Taken together, as a downstream target of the BMP signaling pathway, DLX5 plays a pivotal role in regulating the chondrogenic differentiation of SCAPs. Herein, a rabbit knee cartilage defect model was established to further verify the chondrogenesis capability of DLX5 in SCAPs. In DLX5 over-expressed SCAP transplantation group, the cartilage defects were almost completely repaired by forming new cartilage that was similar in shape and texture to adjacent healthy cartilage. Cartilage staining methods further revealed that the newly regenerated tissues had more glycosaminoglycan and Col2-positive cells. More significantly, the newly formed cartilage showed the typical lacuna-like structure. This evidence proved that the DLX5 accelerated in vivo chondrogenesis of SCAPs.

Many transcription factors are known to dimerize through conserved domains, and studies showed that DLX proteins could form dimeric complexes with other homeobox genes. So, we predicted that DLX5 proteins could interact with other proteins. Previous studies showed that DLX5 proteins appear to have a weak combination with a more divergent homeoprotein, HOXC8 [27]. HOX genes, as a highly conserved subset of the homeobox superfamily, encode several sequence-specific DNA binding proteins acting as transcription factors, which are thought to the specific individual segments of the appendicular skeleton [54]. Studies have shown that by regulating cell cycles and cell adhesion, HOXC8 could regulate the proliferation of chondrocytes and could promote cartilage maturation and endochondral ossification [28, 29]. Therefore, we hypothesized that DLX5 and HOXC8 form a protein complex. Indeed, our Co-IP results showed that DLX5 could combine with HOXC8 to form a protein complex in SCAPs. Previous studies showed that HOX and DLX proteins could function antagonistically because of their ability to form heterodimeric complexes [27]. Cognizant to this, the dynamics of DLX5 and HOXC8 function during chondrogenic differentiation were further evaluated. Then, we investigated the expression of HOXC8 during the chondrogenic differentiation of SCAPs. HOXC8 was found to be upregulated during chondrogenic differentiation in SCAPs. In vitro examinations revealed that it positively regulated the chondrogenic differentiation potential in SCAPs. Furthermore, in vivo studies in the rabbit knee model, HOXC8 promoted SCAP-mediated structure-specific cartilage regeneration. Evidently, these results strongly suggested that HOXC8 enhanced the chondrogenic differentiation potential of SCAPs.

In the same line, RNA-seq analysis demonstrated that LINC01013 was a LncRNA and a downstream gene of HOXC8 and DLX5. It was negatively regulated by HOXC8 and DLX5. HOX genes can recognize and bind to specific sequence motifs (TAAT/ATTA/TTAT/ATAA/TTAC) located in promoter sequences via a conserved helix-turn-helix DNA-binding domain [55]. By bioinformatics analysis, we discovered that the LINC01013 promoter contained abundant specific binding sequences of HOX genes, which might be recognized by HOXC8. As such, the ChIP assay was performed to identify the candidate binding sites of HOXC8. By ChIP assay, we found that HOXC8 could recruit into the LINC01013 promoter, and over-expression of DLX5 could help the recruitment of HOXC8. While in this region, there were three candidate binding elements of HOXC8, ChIP assay could not identify the binding sites accurately. Therefore, further experiments are needed to confirm the specific binding sites.

In recent years, abnormal expression of lncRNAs in bone and cartilage injury in osteoarthritis patients has been reported, suggesting its potential role in the development of osteoarthritis and is a promising target for disease diagnosis and treatment [56]. Some lncRNAs play an important role in the pathogenesis of joint injury and osteoarthritis [57, 58]. The previous study found that LINC01013 enhances invasion of human anaplastic large-cell lymphoma. However, its role in chondrogenic differentiation remained unknown. In the present study, our results showed that LINC01013 negatively regulated the chondrogenic differentiation potential of SCAPs. These indicated that DLX5 and HOXC8 promoted the chondrogenic differentiation by negatively regulating LINC01013 in SCAPs.

## Conclusion

In conclusion, we identified that DLX5 and HOXC8 could enhance the chondrogenic differentiation potential of SCAPs. The downstream target gene of DLX5 and HOXC8, LINC01013, negatively regulated the chondrogenic differentiation potential of SCAPs. Mechanically, DLX5 might apply its regulation in chondrogenic differentiation by interaction with its binding partner, HOXC8, then facilitated HOXC8 to recruit into LINC01013 promoter and inhibited LINC01013 transcript. Our discoveries provided new insights into the underlying mechanism and potential target for promoting the directional differentiation of MSCs and cartilage tissue regeneration.

## Supplementary information

**Additional file 1: Table S1.** Primer sequences used in real-time RT-PCR analysis.

**Additional file 2: Figure S1**. LINC01013 expression in RNA-seq and Schematic diagram of LINC01013 promoter. (A) RNA-seq results showed that the expression of LINC01013 in DLX5 or HOXC8 depleted SCAPs. (B) The diagram of HOXC8 binding element in LINC01013 promoter. Consh, Control shRNA. DLX5sh, DLX5 shRNA. HOXC8sh, HOXC8 shRNA.

## Data Availability

All other relevant datasets have been uploaded as part of additional files.

## Abbreviations

DLX5: Distal-less homeobox 5
HOXC8: Homeobox C8
MSCs: Mesenchymal stem cells
SCAPs: Stem cells from apical papilla
COL2: Collagen II
COL5: Collagen V
SOX9: Sex-determining region Y box protein 9
ALP: Alkaline phosphatase
ChIP: Chromatin immunoprecipitation
GAPDH: Glyceraldehyde-3-phosphate dehydrogenase
BMSCs: Bone marrow stem cells
DPSCs: Dental pulp stem cells
SHEDs: Stem cells from human exfoliated deciduous teeth
PDLSCs: Periodontal ligament stem cells
ICRS: International Cartilage Repair Society
ECM: Extracellular matrix proteins

## References

[1] Martel-Pelletier J, Barr AJ, Cicuttini FM (2016). Osteoarthritis. Nat Rev Dis Primers.

[2] Glyn-Jones S, Palmer AJ, Agricola R (2015). Osteoarthritis. Lancet.

[3] Colombini A, Perucca Orfei C, Kouroupis D (2019). Mesenchymal stem cells in the treatment of articular cartilage degeneration: new biological insights for an old-timer cell. Cytotherapy..

[4] J Jevotovsky DS, Alfonso AR, Einhorn TA, Chiu ES. Osteoarthritis and stem cell therapy in humans: a systematic review. Osteoarthr Cartil 2018;26(6):711–729. 10.1016/j.joca.2018.02.906 29544858

[5] Vega A, Martín-Ferrero MA, Del Canto F (2015). Treatment of knee osteoarthritis with allogeneic bone marrow mesenchymal stem cells: a randomized controlled trial. Transplantation..

[6] Saito MT, Silvério KG, Casati MZ (2015). Tooth-derived stem cells: update and perspectives. World J Stem Cells.

[7] Sharpe PT (2016). Dental mesenchymal stem cells. Development..

[8] Huang GT, Gronthos S, Shi S (2009). Mesenchymal stem cells derived from dental tissues vs. those from other sources: their biology and role in regenerative medicine. J Dent Res.

[9] Yao L, Flynn N (2018). Dental pulp stem cell-derived chondrogenic cells demonstrate differential cell motility in type I and type II collagen hydrogels. Spine J.

[10] Bakopoulou A, Apatzidou D, Aggelidou E (2017). Isolation and prolonged expansion of oral mesenchymal stem cells under clinical-grade, GMP-compliant conditions differentially affects “stemness” properties. Stem Cell Res Ther.

[11] Westin CB, Trinca RB, Zuliani C (2017). Differentiation of dental pulp stem cells into chondrocytes upon culture on porous chitosan-xanthan scaffolds in the presence of kartogenin. Mater Sci Eng C Mater Biol Appl.

[12] Kim H, Park S, Kim K, Ku S, Seo J, Roh S. *Enterococcus faecium* L-15 Cell-Free Extract Improves the Chondrogenic Differentiation of Human Dental Pulp Stem Cells. Int J Mol Sci. 2019;20(3):624. 10.3390/ijms20030624 PMC6386954 30709061

[13] Liu J, Yu F, Sun Y (2015). Concise reviews: characteristics and potential applications of human dental tissue-derived mesenchymal stem cells. Stem Cells.

[14] Murry CE, Keller G (2008). Differentiation of embryonic stem cells to clinically relevant populations: lessons from embryonic development. Cell..

[15] Chen Q, Shou P, Zheng C (2016). Fate decision of mesenchymal stem cells: adipocytes or osteoblasts?. Cell Death Differ.

[16] Stelcer E, Kulcenty K, Rucinski M (2019). The role of microRNAs in early chondrogenesis of human induced pluripotent stem cells (hiPSCs). Int J Mol Sci.

[17] Levi G, Gitton Y (2014). Dlx genes and the maintenance of bone homeostasis and skeletal integrity. Cell Death Differ.

[18] Isaac J, Erthal J, Gordon J (2014). DLX3 regulates bone mass by targeting genes supporting osteoblast differentiation and mineral homeostasis in vivo. Cell Death Differ.

[19] Takechi M, Adachi N, Hirai T (2013). The Dlx genes as clues to vertebrate genomics and craniofacial evolution. Semin Cell Dev Biol.

[20] Stock DW, Ellies DL, Zhao Z (1996). The evolution of the vertebrate Dlx gene family. Proc Natl Acad Sci U S A.

[21] Bendall AJ, Hu G, Levi G, Abate-Shen C (2003). Dlx5 regulates chondrocyte differentiation at multiple stages. Int J Dev Biol.

[22] Ferrari D, Sumoy L, Gannon J (1995). The expression pattern of the distal-less homeobox-containing gene Dlx-5 in the developing chick limb bud suggests its involvement in apical ectodermal ridge activity, pattern formation, and cartilage differentiation. Mech Dev.

[23] Acampora D, Merlo GR, Paleari L (1999). Craniofacial, vestibular and bone defects in mice lacking the distal-less-related gene Dlx5. Development..

[24] Jeffries MA, Donica M, Baker LW (2014). Genome-wide DNA methylation study identifies significant epigenomic changes in osteoarthritic cartilage. Arthritis Rheumatol.

[25] Hsu SH, Noamani B, Abernethy DE (2006). Dlx5- and Dlx6-mediated chondrogenesis: differential domain requirements for a conserved function. Mech Dev.

[26] Ferrari D, Kosher RA (2002). Dlx5 is a positive regulator of chondrocyte differentiation during endochondral ossification. Dev Biol.

[27] Zhang H, Hu G, Wang H (1997). Heterodimerization of Msx and Dlx homeoproteins results in functional antagonism. Mol Cell Biol.

[28] Mucientes A, Herranz E, Moro E, et al. Differential Expression of HOX Genes in Mesenchymal Stem Cells from Osteoarthritic Patients Is Independent of Their Promoter Methylation. Cells. 2018;7(12):244. 10.3390/cells7120244 PMC6316585 30563049

[29] Kruger C, Kappen C (2010). Expression of cartilage developmental genes in Hoxc8- and Hoxd4-transgenic mice. PLoS One.

[30] Yueh YG, Gardner DP, Kappen C (1998). Evidence for regulation of cartilage differentiation by the homeobox gene Hoxc-8. Proc Natl Acad Sci U S A.

[31] Razmara E, Bitaraf A, Yousefi H (2019). Non-coding RNAs in cartilage development: an updated review. Int J Mol Sci.

[32] Sun H, Peng G, Ning X, Wang J, Yang H, Deng J (2019). Emerging roles of long noncoding RNA in chondrogenesis, osteogenesis, and osteoarthritis. Am J Transl Res.

[33] Yang H, Liang Y, Cao Y, Cao Y, Fan Z. Homeobox C8 inhibited the osteo-/dentinogenic differentiation and migration ability of stem cells of the apical papilla via activating KDM1A. J Cell Physiol. 2020;10.1002/jcp.29687. 10.1002/jcp.29687 32246725

[34] Yang H, Fan J, Cao Y (2019). Distal-less homeobox 5 promotes the osteo-/dentinogenic differentiation potential of stem cells from apical papilla by activating histone demethylase KDM4B through a positive feedback mechanism. Exp Cell Res.

[35] Wang KH, Wan R, Chiu LH (2018). Correction: Effects of collagen matrix and bioreactor cultivation on cartilage regeneration of a full-thickness critical-size knee joint cartilage defects with subchondral bone damage in a rabbit model. PLoS One.

[36] Chen Z, Yan C, Yan S (2018). Non-invasive monitoring of in vivo hydrogel degradation and cartilage regeneration by multiparametric MR imaging. Theranostics..

[37] Hoemann C, Kandel R, Roberts S (2011). International Cartilage Repair Society (ICRS) recommended guidelines for histological endpoints for cartilage repair studies in animal models and clinical trials. Cartilage..

[38] Mainil-Varlet P, Van Damme B, Nesic D (2010). A new histology scoring system for the assessment of the quality of human cartilage repair: ICRS II. Am J Sports Med.

[39] Zhang R, Ma J, Han J (2019). Mesenchymal stem cell related therapies for cartilage lesions and osteoarthritis. Am J Transl Res.

[40] Li X, Wang M, Jing X (2018). Bone marrow- and adipose tissue-derived mesenchymal stem cells: characterization, differentiation, and applications in cartilage tissue engineering. Crit Rev Eukaryot Gene Expr.

[41] Solchaga LA, Penick K, Goldberg VM (2010). Fibroblast growth factor-2 enhances proliferation and delays loss of chondrogenic potential in human adult bone-marrow-derived mesenchymal stem cells. Tissue Eng Part A..

[42] Nemeth CL, Janebodin K, Yuan AE (2014). Enhanced chondrogenic differentiation of dental pulp stem cells using nanopatterned PEG-GelMA-HA hydrogels. Tissue Eng Part A.

[43] Jensen J, Tvedesøe C, Rölfing JH (2016). Dental pulp-derived stromal cells exhibit a higher osteogenic potency than bone marrow-derived stromal cells in vitro and in a porcine critical-size bone defect model. SICOT J.

[44] Moshaverinia A, Xu X, Chen C (2013). Dental mesenchymal stem cells encapsulated in an alginate hydrogel co-delivery microencapsulation system for cartilage regeneration. Acta Biomater.

[45] Longoni A, Utomo L, van Hooijdonk IE (2020). The chondrogenic differentiation potential of dental pulp stem cells. Eur Cell Mater.

[46] Karaöz E, Demircan PC, Sağlam O (2011). Human dental pulp stem cells demonstrate better neural and epithelial stem cell properties than bone marrow-derived mesenchymal stem cells. Histochem Cell Biol.

[47] Ruscitto A, Morel MM, Shawber CJ (2020). Evidence of vasculature and chondrocyte to osteoblast transdifferentiation in craniofacial synovial joints: implications for osteoarthritis diagnosis and therapy. FASEB J.

[48] Wu JJ, Weis MA, Kim LS (2009). Differences in chain usage and cross-linking specificities of cartilage type V/XI collagen isoforms with age and tissue. J Biol Chem.

[49] Barter MJ, Gomez R, Hyatt S (2017). The long non-coding RNA ROCR contributes to SOX9 expression and chondrogenic differentiation of human mesenchymal stem cells. Development..

[50] Lefebvre V, Angelozzi M, Haseeb A (2019). SOX9 in cartilage development and disease. Curr Opin Cell Biol.

[51] Chijimatsu R, Saito T (2019). Mechanisms of synovial joint and articular cartilage development. Cell Mol Life Sci.

[52] Murphy MK, Huey DJ, Hu JC, Athanasiou KA (2015). TGF-β1, GDF-5, and BMP-2 stimulation induces chondrogenesis in expanded human articular chondrocytes and marrow-derived stromal cells. Stem Cells.

[53] Nishimura R, Hata K, Ikeda F (2008). Signal transduction and transcriptional regulation during mesenchymal cell differentiation. J Bone Miner Metab.

[54] Krumlauf R (1994). Hox genes in vertebrate development. Cell..

[55] Ruthala K, Gadi J, Lee JY (2011). Hoxc8 downregulates Mgl1 tumor suppressor gene expression and reduces its concomitant function on cell adhesion. Mol Cells.

[56] Fu M, Huang G, Zhang Z (2015). Expression profile of long noncoding RNAs in cartilage from knee osteoarthritis patients. Osteoarthr Cartil.

[57] Huang MJ, Zhao JY, Xu JJ (2019). lncRNA ADAMTS9-AS2 controls human mesenchymal stem cell chondrogenic differentiation and functions as a ceRNA. Mol Ther Nucleic Acids.

[58] Liu Q, Zhang X, Dai L (2014). Long noncoding RNA related to cartilage injury promotes chondrocyte extracellular matrix degradation in osteoarthritis. Arthritis Rheumatol..

