# Supplementary material for: DLX5 and HOXC8 enhance the chondrogenic differentiation potential of stem cells from apical papilla via LINC01013
Source: Stem Cell Res Ther. 2020 Jul 6;11:271. doi: 10.1186/s13287-020-01791-8 (PMC7336658; doi:10.1186/s13287-020-01791-8)
Supplement: Supplementary file 1 — Additional file 1: Table S1. Primer sequences used in real-time RT-PCR analysis. [file 13287_2020_1791_MOESM1_ESM.docx]

**Supplementary Table 1. Primers sequences used in the real-time RT-PCR**

| **Gene Symbol** | **Primer Sequences (5’—3”)** |
| --- | --- |
| GAPDH-F | CGGACCAATACGACCAAATCCG |
| GAPDH-R | AGCCACATCGCTCAGACACC |
| HOXC8-F | ACCGGCCTATTACGACTGC |
| HOXC8-R | TGCTGGTAGCCTGAGTTGGA |
| COL5-F | GTGGCACAGAATTGCTCTCA |
| COL5-R | AAACACGATGATGCCATTGA |
| SOX9-F | CCCTTCAACCTCCCACACTA |
| SOX9-R | TGGTGGTCGGTGTAGTCGTA |
| COL2-F | CATCCCACCCTCTCACAGTT |
| COL2-R | TCTGCCCAGTTCAGGTCTCT |
| DLX5-F | TTCCAAGCTCCGTTCCAGAC |
| DLX5-R | GAATCGGTAGCTGAAGACTCG |
| LINC01013-F | CATCCAGCAGTGCCCAAGTA |
| LINC01013-R | GCTCTCCTTTGTTCCAGGCT |
