# Supplementary figures and images for: DLX5 and HOXC8 enhance the chondrogenic differentiation potential of stem cells from apical papilla via LINC01013
Source: Stem Cell Res Ther. 2020 Jul 6;11:271. doi: 10.1186/s13287-020-01791-8 (PMC7336658; doi:10.1186/s13287-020-01791-8)

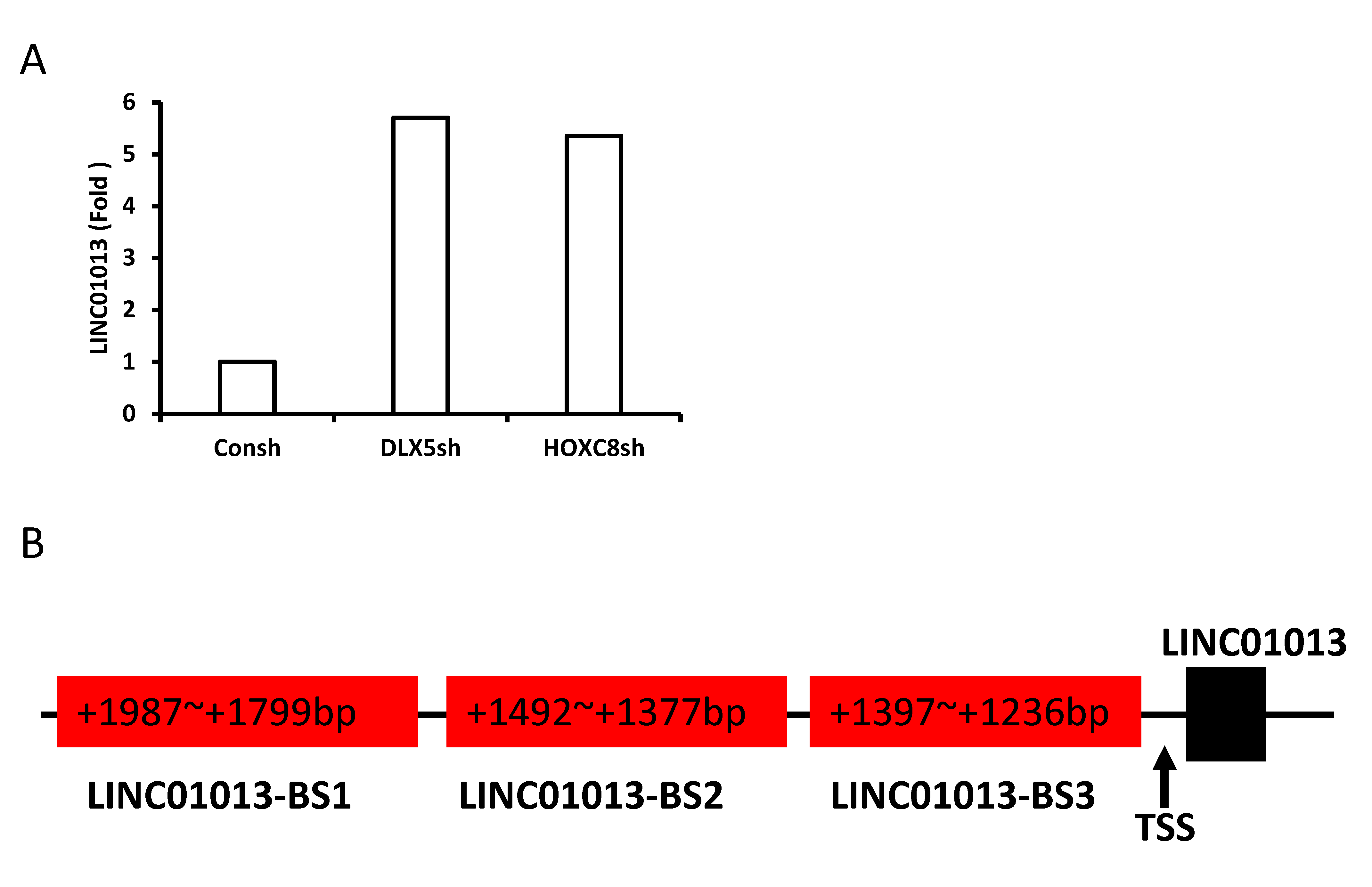

Supplement: Supplementary file 2 — Additional file 2: Figure S1. LINC01013 expression in RNA-seq and Schematic diagram of LINC01013 promoter. (A) RNA-seq results showed that the expression of LINC01013 in DLX5 or HOXC8 depleted SCAPs. (B) The diagram of HOXC8 binding element in LINC01013 promoter. Consh, Control shRNA. DLX5sh, DLX5 shRNA. HOXC8sh, HOXC8 shRNA. [file 13287_2020_1791_MOESM2_ESM.tiff]
